# A Random Forest approach to identify metrics that best predict match outcome and player ranking in the esport Rocket League

**DOI:** 10.1038/s41598-021-98879-9

**Published:** 2021-09-29

**Authors:** Tim D. Smithies, Mark J. Campbell, Niall Ramsbottom, Adam J. Toth

**Affiliations:** 1 Department of Physical Education & Sport Science, University of Limerick, Castletroy, Limerick, Ireland; 2 Lero, The Science Foundation Ireland Research Centre for Software, University of Limerick, Castletroy, Limerick, Ireland

**Keywords:** Statistics, Scientific data, Human behaviour

## Abstract

Notational analysis is a popular tool for understanding what constitutes optimal performance in traditional sports. However, this approach has been seldom used in esports. The popular esport “Rocket League” is an ideal candidate for notational analysis due to the availability of an online repository containing data from millions of matches. The purpose of this study was to use Random Forest models to identify in-match metrics that predicted match outcome (performance indicators or “PIs”) and/or in-game player rank (rank indicators or “RIs”). We evaluated match data from 21,588 Rocket League matches involving players from four different ranks. Upon identifying goal difference (GD) as a suitable outcome measure for Rocket League match performance, Random Forest models were used alongside accompanying variable importance methods to identify metrics that were PIs or RIs. We found *shots taken, shots conceded, saves made,* and *time spent goalside of the ball* to be the most important PIs, and *time spent at supersonic speed*, *time spent on the ground*, *shots conceded* and *time spent goalside of the ball* to be the most important RIs. This work is the first to use Random Forest learning algorithms to highlight the most critical PIs and RIs in a prominent esport.

## Introduction

The popularity of esports (competitive organised video game play) has grown rapidly over the past ten years to the point where viewership now rivals that in many traditional sports. In fact, it has been estimated that over one billion individuals viewed esports content in 2020^1^. This rapid rise in interest in esports has led to increasing professionalisation, investment and attention towards optimising performance among the top players with the ultimate goal of individual or team success. However, extremely little research exists to date exploring what constitutes optimal performance within various esports. Until the factors that determine optimal performance are understood within a given esport (or any activity more broadly), it is very difficult to create and implement effective and efficient strategies towards achieving optimal performance.

For most tasks, optimizing performance is often predicated on the identification of performance indicators (PIs; individual variables that predict the overall outcome of a match or performance). A very popular approach to identifying PIs is a “notational approach”. Notational analyses are used to study of patterns within a match/contest/competition/performance that lead to a successful overall outcome^2^ and can uncover the components most important for match outcome. In traditional sports, identifying the PIs most important for successful task performance helps players and coaches to better direct focus to those key components to accelerate learning and, ultimately, improve performance. Thus, in traditional sport research, many have employed a notational approach to identify PIs in Australian Rules Football^3^, basketball^4,5^, ice hockey^6^, rugby league^7,8^, and rugby union^9–14^.

By using notational analysis to understand the components of an activity that are most important to success, one can direct their attention to those components to accelerate learning and ultimately improve performance. An example of a training method that could benefit from this understanding is Variable Priority Training (VPT), in which individuals complete a task with focused attention specifically towards improving key PIs within the task^15^. VPT has been demonstrated to enhance learning in video game contexts when compared to Fixed Priority Training (FPT: focussing on all aspects of a task)^15,16^.

In light of the evidence above, notational analyses may stand to benefit esports. Notational analysis could be used in a similar way to that in the traditional sport examples mentioned above to find the most important PIs within a given game to focus on, resulting in more efficient training and use of techniques such as VPT to improve esport performance. However, little research has explored this topic in esports to date. One recent study has started to identify the important PI’s for differentiating expertise in the First Person Shooter (FPS) esport, CS:GO, which has informed commercially available training software^17^, while two others have identified PIs in Multiplayer Online Battle Arena (MOBA) esports^18,19^. The lack of notational analysis and subsequent analysis in esports is surprising given that esports appear ideal for such analyses as they are played digitally, with the ability to store in-game metrics directly for any game. However similarly to “traditional sports”, esports are extremely diverse in game mechanics, objectives, equipment, and team size and structure, meaning that PIs from one esport are unlikely to be relevant to another. Additionally, in-match data can be difficult to obtain as they are often not made available by game development companies.

One such esport whereby performance data are readily available, making it an ideal candidate for notational analyses, is Rocket League. Rocket League is a “vehicular soccer video game” released in 2015 by Psyonix. In Rocket League, players each control a rocket-powered vehicle with the goal of hitting a large ball into a goal that is similar to a football/soccer goal, while simultaneously defending their own goal. The popularity of Rocket League has rapidly escalated since it became free-to-play on September 27, 2020, with its peak concurrent player count of 1.85 million surpassing the popular esport mainstay, CS:GO, by more than 500,000^20,21^. Alongside this high concurrent player count, Rocket League has reported ~ 90 million monthly users every month since November 2020^22^, approximately triple that received for CS:GO^23^. Additionally, Rocket League has a thriving esports scene, with competing teams from top esports organisations such as Team Liquid, G2 esports, and NRG esports, and with ~ 12 million USD won through Rocket League competition (As of 12/03/2021; Esports Earnings^24^). Overall, its popularity, the drive for optimising player performance at the top levels and the wealth of freely and readily available match data position Rocket League as an ideal candidate for notational analysis and the identification of the PIs that predict performance outcomes in this esport.

In Rocket League, players can save match replays and upload them to “www.ballchasing.com”, which in turn makes over 65 in-match metrics publicly available. As of May 5, 2021, there are over 24.5 million match replays available on the “www.ballchasing.com” online repository, across matches of various formats and with players of various ranks, freely available for anyone to download. Such volume of readily available match data is unheralded in esports and in traditional sports.

Previous research has employed the use of general linear mixed effects models^18^ or solitary classification and regression trees (CARTs)^19^ for PI identification within esports. While these methods have their benefits, one superior approach that has yet to be adopted in esports is the use of Random Forest models^25^. Random Forests are a machine learning ensemble algorithm and refer to an ensemble of CARTs each trained using a unique bootstrapped data set and random selection of splitting predictor features. Each case in the original data set is then run through all CARTs in the forest for which it was not part of the training process (and hence is “out-of-bag” or “OOB” for these CARTs), and the mean (for a regression model) or modal (for a classification model) response is considered the overall response of the model for that case.

Random Forests are a superior option to linear or logistic models and solitary CARTs for the current data and objectives for many reasons. Firstly, Random Forests can incorporate non-linear effects, and are superior to alternate methods at modelling complex interactions when the interactions are not, or cannot be, pre-specified^26^. This is ideal given the exploratory nature of PI identification in esports research and the unknown properties of the metrics included in model creation. Moreover, Random Forests have no distributional assumptions for predictor or response variables and are thus resistant to bias from non-parametric data, skewed data, and even nominal data, and perform exceptionally well even when many predictors are weak (or “noise”)^25–27^. Moreover, the fact that Random Forests are an amalgamation of many CARTs using a bootstrapped data samples and a random selection of predictor variables for node splitting per tree, they inherently provide much greater predictive ability and reduce propensity for overfitting when compared to the CART method alone^25,28^, making them suitable for large datasets. Given the above advantages over existing methods and that Random Forest have been used previously to identify PIs within traditional sports^7–10,13^ they are arguably the most optimal method to identify PIs in Rocket League and esports more broadly.

By leveraging the immense amount of freely available match data in Rocket League and utilising the state-of-the-art notational approach of Random Forest machine learning modelling, the purpose of this study is to identify metrics that predict performance (PIs) and expertise (RIs) within the esport, Rocket League. Specifically, we aimed to first identify a suitable match outcome measure that could capture more information than provided by binary win vs. loss. We then aimed to identify in-match metrics that best predict our match outcome measure, across a variety of player ability levels. Finally, we aimed to also identify in-match metrics that best predict the ability level of the players within matches themselves.

## Results

While Rocket League can be played either individually (1v1) or in teams of two or three, the analyses of multiplayer competition requires consideration of the interactions between teammates, which is a necessary factor for team sports/esports and can greatly complicate analyses^29^. Therefore, this study focused solely on 1v1 Rocket League, which benefits from the fact that match metrics in this format are a direct result of player actions or interactions between player and opponent.

**Figure 1:**
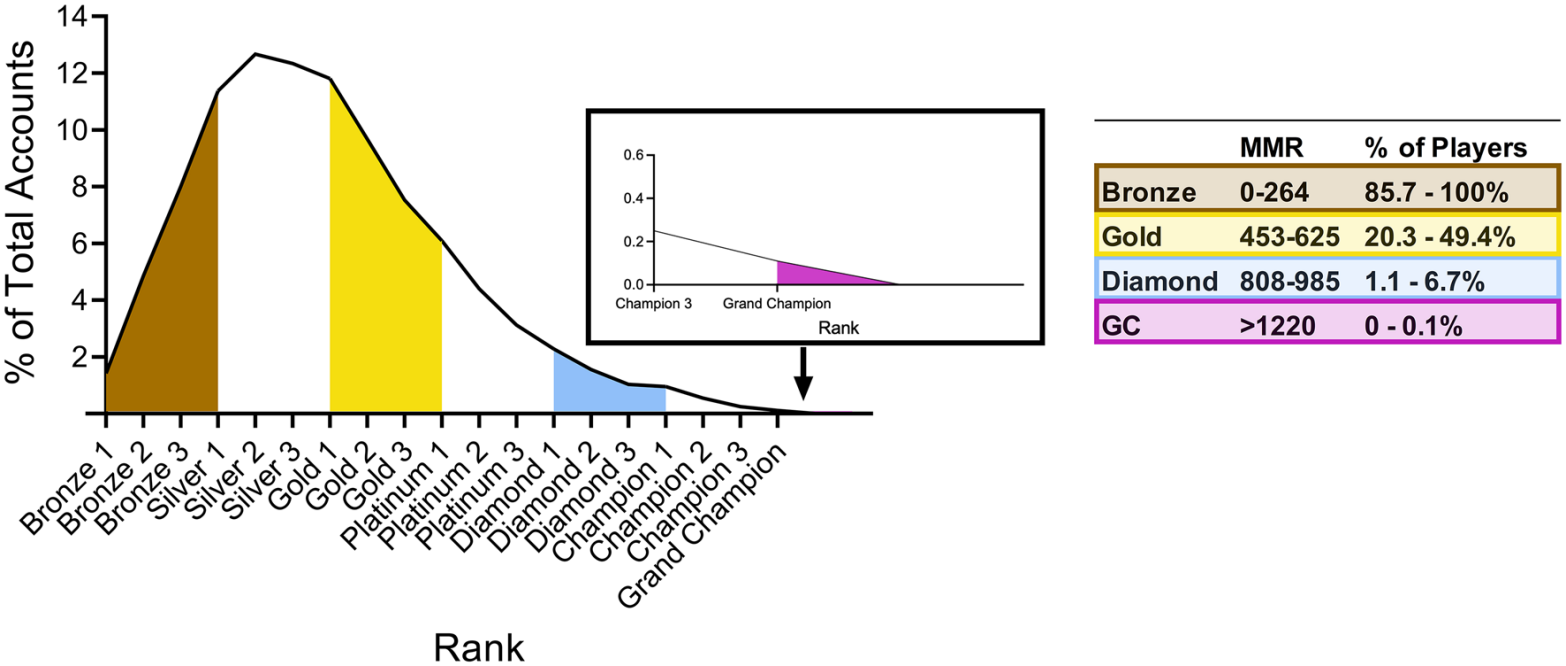
A density plot showing the distribution of accounts within the Rocket League rank system. Colour shaded areas correspond to the skill brackets, and associated MMRs, considered for the current study. This distribution is as per season 14 of Rocket League, which was the season at the time of the most recent match used in the analysis.

The data from four Rocket League rank groupings were considered for our analyses: Bronze, Gold, Diamond, and Grand Champion (GC). These rank groups were chosen to allow for the capture of a broad range of ability levels while simultaneously creating clear distinctions between each rank group (see Fig. 1). Ranks within Rocket League correspond to a player’s matchmaking rating (MMR). A players MMR increases after every win and decreases after every loss, with the magnitude of the increase/decrease determined by the difference between players’ MMR before the match.

### Obtaining a continuous performance outcome measure

In line with the first aim of this study, we determined a continuous match outcome metric that that could be reasonably substituted for the binary win vs. loss (WL) outcome measure while providing additional information regarding the severity of a win or loss. To do this, the *in-game score difference* (IGSD) and *goal difference* (GD) metrics were considered as candidates.

**Figure 2:**
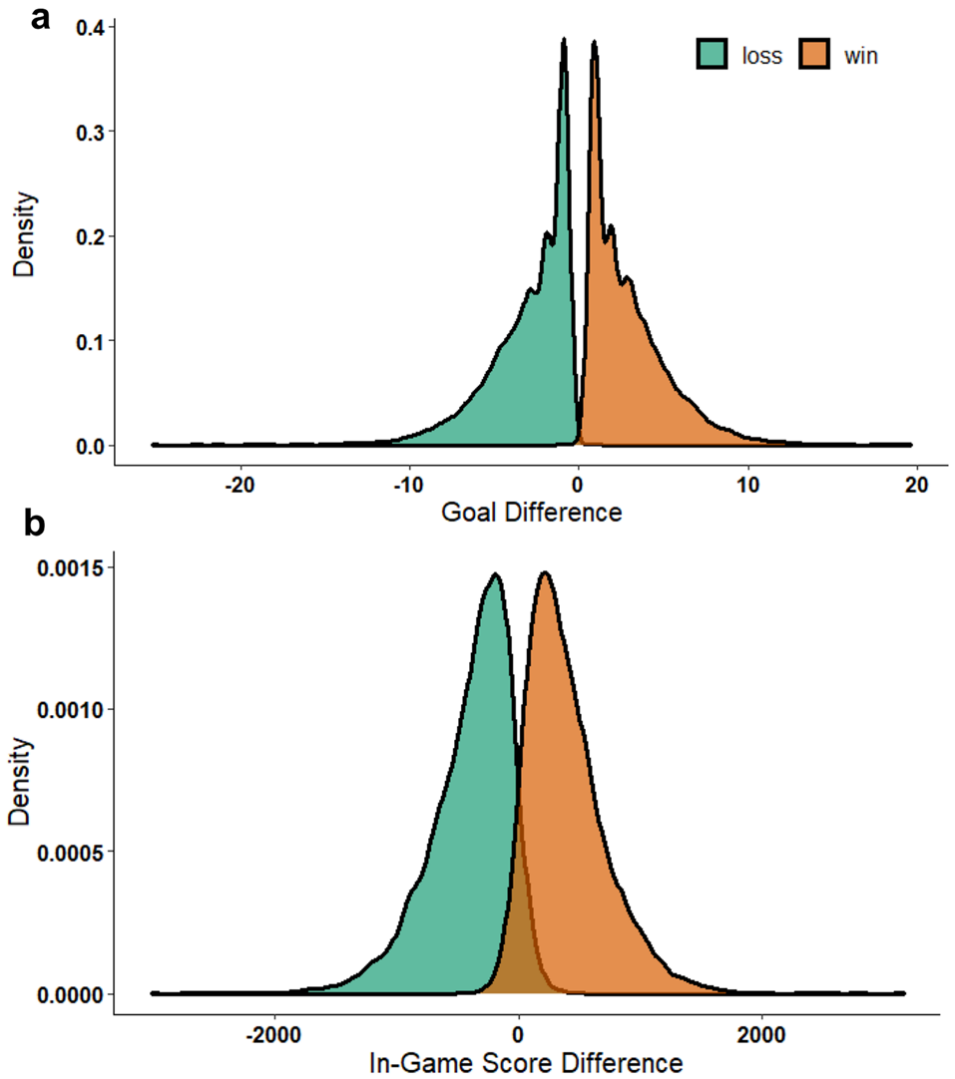
Density plots showcasing (**a**) the distributions of goal difference and (**b**) in-game score difference as a function of win vs. loss.

We conducted point-biserial correlations that tested the association between each metric and WL across all matches and across matches within each specific rank group. All point-biserial correlations demonstrated large (rpb > 0.70) significant (p < 0.001) associations, however GD yielded larger association with WL for matches within each rank and when matches for all ranks were combined (Bronze: r = 0.77, Gold: r = 0.80, Diamond: r = 0.79, GC: r = 0.78, all ranks: r = 0.79) compared to IGSD (Bronze: r = 0.76, Gold: r = 0.78, Diamond: r = 0.77, GC: r = 0.75, all ranks: r = 0.77). Finally, we noted that when using zero as a cut-off for IGSD and GD (positive scores corresponding to “win”, and negative scores corresponding to “loss”), IGSD correctly identified wins 93.56% of the time, and losses 93.70% of the time, while GD correctly identified wins and losses 99.94% of the time. Figure 2 displays the distribution of the data from all skill brackets combined using a density plot (default bandwidth).

Through these analyses, we demonstrate that GD and IGSD are both appropriate continuous variables for game outcome. However, due to the superior association of GD with WL across all matches and matches within each rank group, GD was used as the performance outcome measure in subsequent analyses.

### Obtaining indicators of performance (PIs)

Random Forest regression models were created using the raw-score metrics (player metrics, not accounting for opponent) and difference-score metrics (player metrics accounting for opponent). These models were created for 1v1 Rocket League matches occurring in Bronze rank (lowest in-game rank; 2527 matches), Gold rank (7226 matches), Diamond rank (7193 matches) and Grand Champion (GC) rank (highest in-game rank; 4642 matches), as well as in all matches regardless of rank (21,588 matches). The match outcome variable for these regression models was the goal difference (GD) between players within a match. These models were used to identify the in-game metrics that best predicted in match outcome and could thus be described as PIs for Rocket League.

All of the models created were highly predictive of GD (R^2^ > 0.7). As can be seen in Table 1, models created using the difference-scores were better able to predict match outcome compared to models using raw-scores in all cases.

**Table 1:** R^2^ (mean of squared residuals) of the Random Forest models created using the raw and difference-score metrics for each ranks and for all ranks combined.

	Bronze	Gold	Diamond	GC	All
Raw-score	0.793 (3.91)	0.741 (3.55)	0.725 (3.66)	0.713 (4.06)	0.747 (3.61)
Difference-score	0.841 (2.99)	0.823 (2.43)	0.823 (2.35)	0.816 (2.59)	0.839 (2.29)

#### Raw-score models

**Figure 3:**
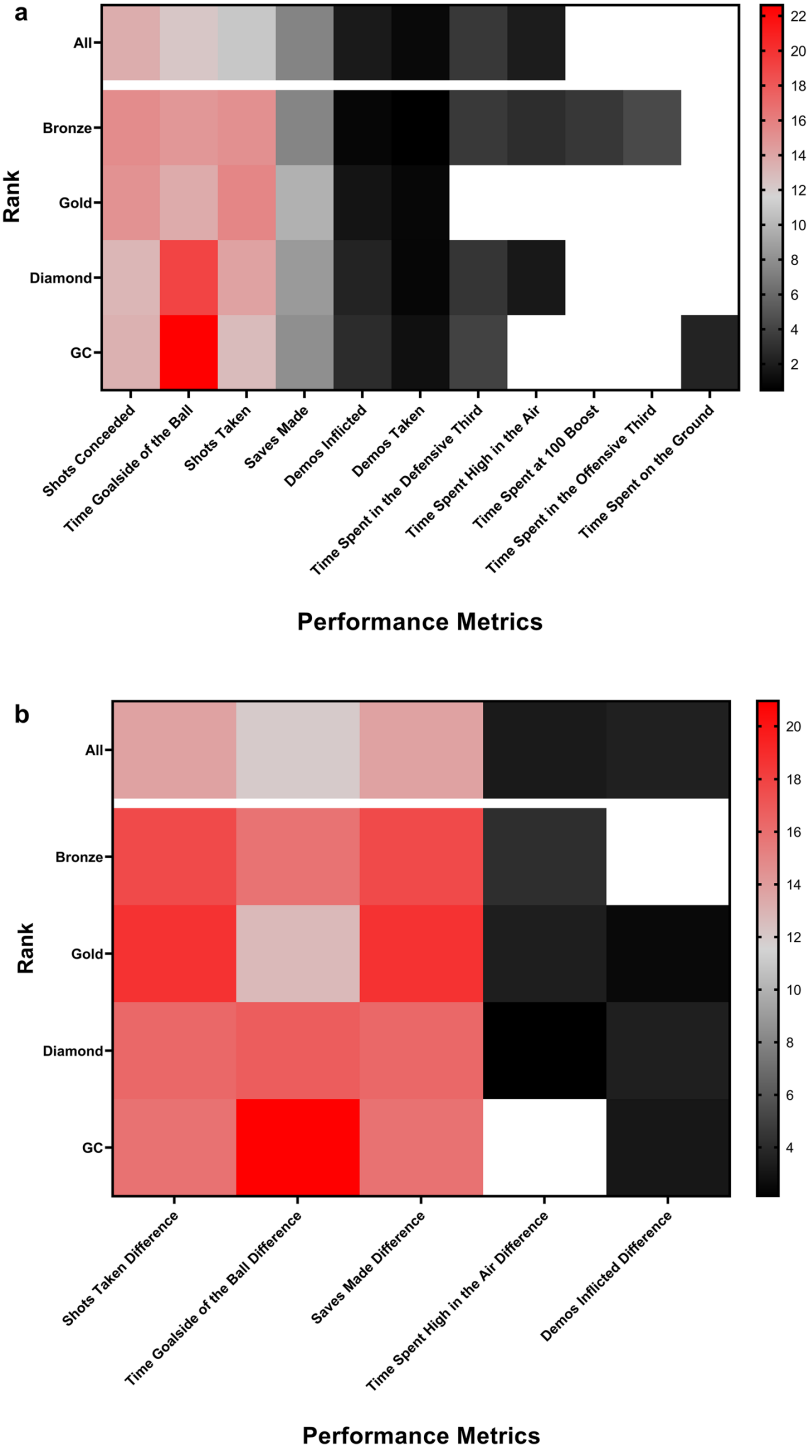
Heat map displaying the percentage of the total increase in MSE that can be found when a metric is permuted individually compared to the sum of increase in MSE for all metrics when permuted individually. Only metrics that were significantly important for predicting GD within each raw-score and difference-score model are presented. White squares represent metrics that were not significant for the rank they are assigned to. (**a**) Results from raw-score regression models, and (**b**) results from difference-score regression models.

In all Random Forest regression models using raw-score metrics, the following metrics led to a significant (*p* < 0.05) increase in mean square error (MSE) when permuted, and hence were identified as PIs: *shots taken*, *shots conceded*, *time spent goalside of the ball*, *saves made*, *demos taken*, and *demos inflicted*. Figure 3a shows the relative contribution that each PI metric made to the total MSE increase when all PIs were included together for matches within each rank category, as well as for matches across all ranks combined, for the raw-score models. For matches in the Bronze rank, Gold rank, and when all ranks are considered, *shots taken* and *shots conceded* were more important than *time spent goalside of the ball*, whereas for matches in the Diamond rank and GC rank, *time spent goalside of the ball* was more important than *shots taken,* and *shots conceded*.

#### Difference-score models

In all Random Forest regression models using difference-score metrics, the following metrics led to a significant (*p* < 0.05) increase in MSE when permuted, and were hence classified as PIs: *shots taken difference*, *time spent goalside of the ball difference*, and *saves made difference*. Figure 3b shows the relative contribution that each PI makes to the total MSE increase when all PIs are included together for matches within each rank category, as well as for matches across all ranks combined, for the difference-score models. For matches in the Bronze rank, Gold rank, and when all ranks are considered, *shots taken difference* and *shots conceded difference* were more important than *time spent goalside of the ball difference*, whereas for matches in the Diamond rank and GC rank, *time spent goalside of the ball difference* was more important than *shots taken difference* and *shots conceded difference*. *Saves made difference* was also more important than *time spent goalside of the ball difference* for matches in the Gold rank and when all ranks are considered.

### Obtaining indicators of in-game rank (RIs)

The Random Forest classification model correctly classified the rank of players within 1764 of 2527 Bronze matches (69.81%), 5394 of 7226 Gold matches (74.65%), 5098 of 7193 Diamond matches (70.87%), and 3417 of 4642 GC matches (73.61%), resulting in an overall out-of-bag (OOB) accuracy of 72.6%.

**Figure 4:**
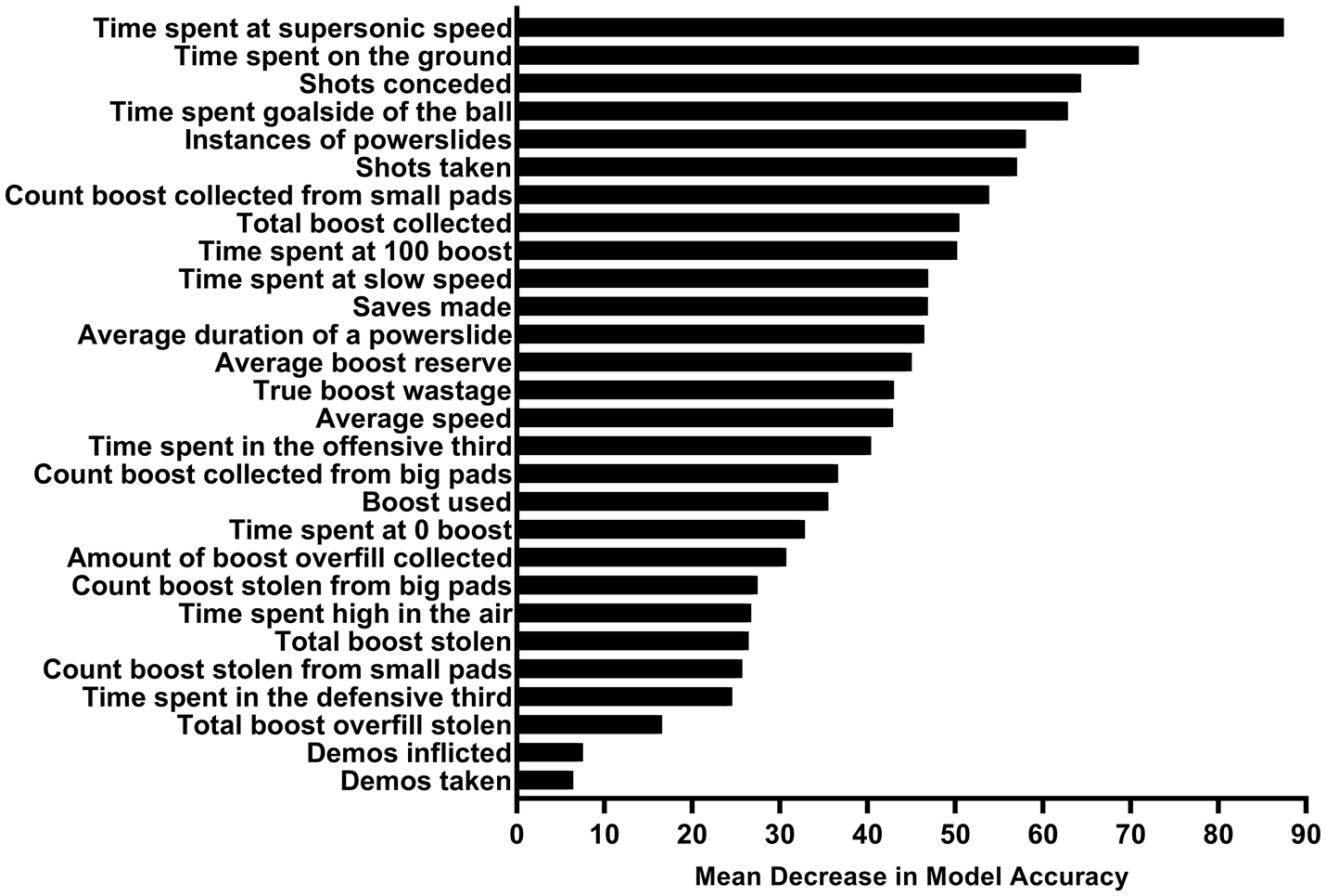
Metrics found to be of significant importance to the classification model created to predict the ranks of individuals playing 1v1 Rocket League, ordered by the increase in mean decrease in accuracy experienced within the model when each metric was permuted.

All metrics were found to significantly decrease the accuracy of the model when permuted (*p* < 0.05), and so were deemed RIs in Rocket League (Fig. 4).

**Figure 5:**
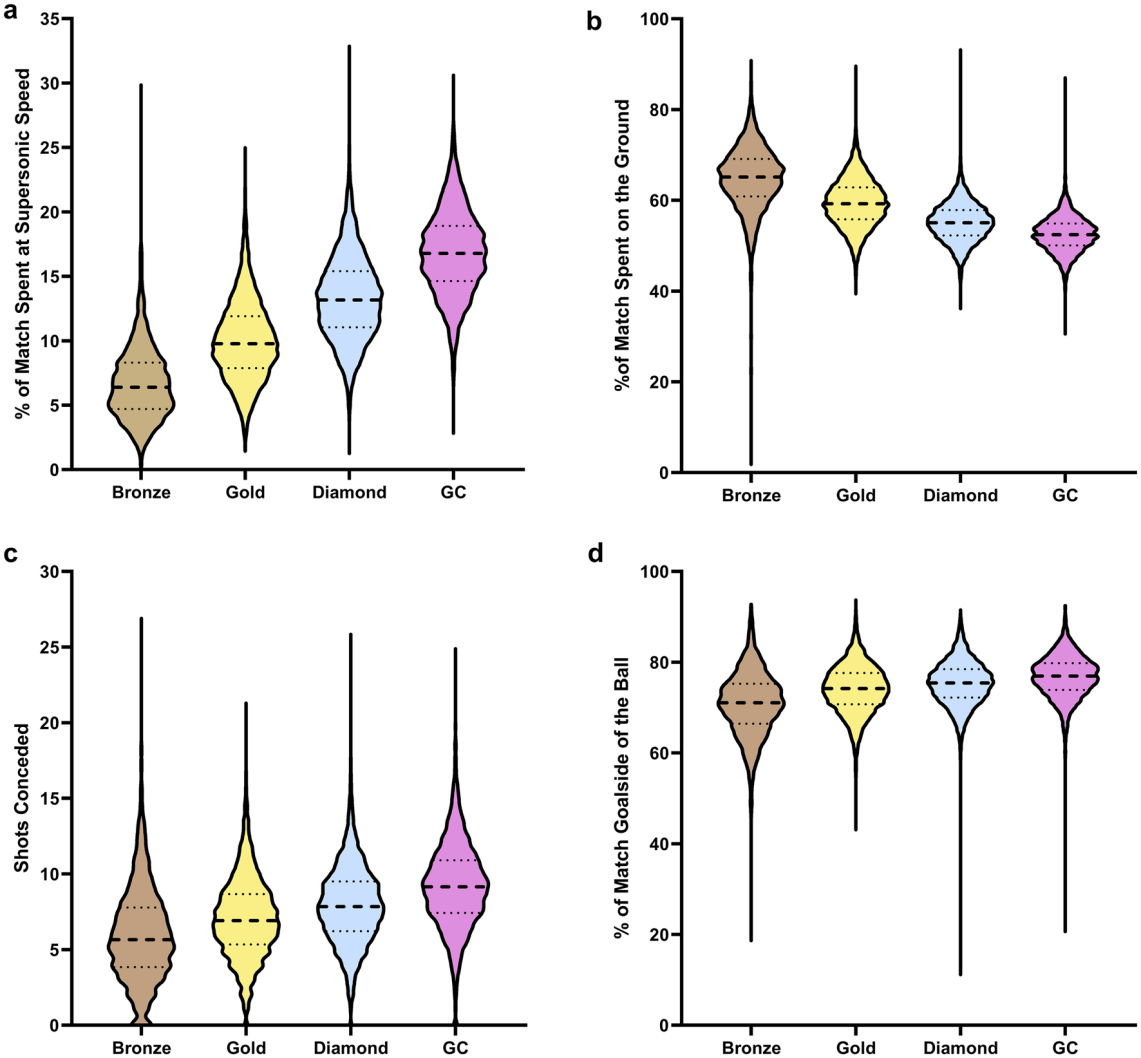
Violin plots displaying the means and distributions, within each rank, of the four most important features for predicting rank, (**a**) time spent at supersonic speed, (**b**) time spent on the ground, (**c**) shots conceded, (**d**) time spent goalside of the ball.

Overall, *time spent at supersonic speed*, *time spent on the ground*, *shots conceded*, and *time spent goalside of the ball* were the four RIs most important to the Random Forest model for correctly classifying data according to the rank of the players within the match. Violin plots showing the means and distributions of these four RIs across included ranks are displayed in Fig. 5.

## Discussion

In this work, we used a Random Forest machine learning algorithm analysis to identify key performance metrics that predicted expertise (RIs) and match outcome (PIs) for the first time in a prominent esport, Rocket League. Specifically, we first aimed to identify a continuous match *outcome* metric that provided more information on in-game performance than the binary win vs. loss measure. Goal difference (GD) was identified as this suitable match outcome metric. Secondly, we aimed to identify metrics that are significantly important to influencing our outcome metric, GD (labelled as “PIs”), both across matches played by players of specific ranks and across matches played by those of all ranks. Hence, these PIs differentiate good and poor performance *within* a given rank. Here, we found specific PIs that are important to GD across all ranks, as well as PIs that are only important to GD in matches with players of specific ranks. Thirdly, we aimed to identify metrics that best predicted player expertise or rank within matches (labelled as “RIs”). These RIs differentiate *between* players of different ranks. All metrics were significantly important to the classification of rank in our Random Forest classification model. Importantly, we show for the first time the order of importance that each metric has for the prediction of rank within our model, with *time at supersonic speed*, *time spent on the ground*, *shots conceded*, and *time spent goalside of the ball* being the four that decreased the performance of the model the greatest when permuted. The following discusses the implications of these findings.

Firstly, our finding that difference-score metrics lead to better match outcome prediction compared to raw-score metrics corroborates previous literature in rugby union^9^. Models incorporating difference-score metrics for any rank were able to account for over 80% of the variance in GD between two players in a given match. This highlights the utility of the in-game statistics obtained from the online repository “www.ballchasing.com” for Rocket League and the utility of Random Forest models for predicting performance within Rocket League.

Focussing on PIs within the difference-score models, when compared to a rank-matched opponent, taking more shots, making more saves, and spending more time goalside of the ball all appear to be beneficial for success in Rocket League matches, regardless of one’s rank. The difference in *time spent goalside of the ball* was found to be most important within higher ranked matches (Diamond & GC), suggesting that as the quality of players increases, so does the relative importance of maintaining one’s positioning between the ball and one’s own goal, compared to simply taking more shots or making more saves. This could be due to the greater ability of higher ranked players to swiftly and accurately shoot a ball into a goal left unattended due to an opponent’s poor positioning. This also may suggest that higher ranked players may generally be well served to adopt a “safer” playstyle, reducing the number of high-risk attacking plays such as “air dribbles” (referring to when a player achieves many controlled touches on the ball while both the player and ball are in the air) that if failed, might leave them positioned in front of the ball. For GC rank players, this is further supported by the finding that increasing time spent high in the air (a necessity for “air dribbles”) did not predict performance, whereas this was a PI of match outcome in all other ranks.

When considering the application this new awareness of important PIs, Rocket League players of all ranks can leverage Variable Priority Training (VPT), which has already been demonstrated to be superior to Fixed Priority Training (FPT)^15^, to actively focus on improving performance on the key metrics that are actually shown to be important for match outcome. Based on the results, a player might specifically work to improve spending more time goalside of the ball than their opponent during their matches. Our results also suggest that lower ranked players (Bronze and Gold) could harness VPT by monitoring the shots they take relative to their opponent during matches and focussing on skills that facilitate improvement on this PI. This has been discussed by professional Rocket League coaches previously as a beneficial strategy as similarly lower-ranked opponents are less likely to save shots regardless of quality^30^. Inflicting more demos on your opponent than they do on you also appears to provide a performance benefit in matches for all ranks except those in the Bronze rank group. A demo (short for demolition) is achieved when one player drives into an opposing player at supersonic speed at the correct angle and removes them from the field for three seconds before the opposing player respawns in one of two prespecified locations in their defensive third. The fact that this metric was not found to be a PI for Bronze level matches may be due to the fact that Bronze players may not possess the skills to capitalise on the three second advantage awarded by a demo to score, whereas higher ranked players may be better able to use demos to score or prevent goals.

The metrics that best predicted differences between ranks (RIs) were not necessarily predictive of performance when two rank-matched players play against one another (i.e., within rank). For example, the percentage of time that a player spent at supersonic speed was the most important RI (Fig. 5), whereas this metric it did not significantly improve the ability of regression models to predict the outcome of a match within any given rank group. The fact that *time at supersonic speed* was found to be a RI and not a PI may be due to the fact that playing at higher ranks requires one to have the ability to play at near maximum speed for longer durations so as to match the speed of the opponent in case they were to attack at maximum speed. However, once both players are able to do so, attacking at supersonic speed does not provide additional benefit within the match between two similarly ranked players. This explanation can also be applied to the PI *number of powerslides*, which, when permuted from the model, led to a large decrease in accuracy (which demonstrates its high importance) in the classification model predicting rank. Powerslides are a difficult manoeuvre that provide the opportunity to maintain speed when landing on the ground and turning sharply, however powerslide turns can be difficult to control. Higher skilled players appear to use this mechanic more often to achieve greater control of their car, however when players are of similar rank, powersliding more or less than an opponent within a match does not appear to provide an advantage. Taken together, higher rank players show better control over the movement of their car and are able to play a greater proportion of their matches at high speed. However, within rank-matched matches, this does not predict match outcome. Therefore, our findings suggest that while focussing on game speed and car movement may not provide immediate benefit to the outcome within matches, these PIs are important to develop as they may facilitate one’s improvement in overall expertise over time.

### Significance

While the identification of PIs to predict match outcome and in-game ranking within Rocket League provides new knowledge regarding how Rocket League players and coaches may structure training programs, the results from this analysis are also foundational for future experimental work utilising esports as a performance arena. Esports have been identified as a promising new avenue to study expertise^31^, due to their data rich nature, continuous and accurate skill rating systems (Elo), and the naturally controlled, laboratory like environment that esports are typically engaged in. More recently, esports have been identified as an ideal framework for exploring whether task expertise moderates task performance deficit experienced from sleep loss^32^, with applications spanning beyond esports due to the shared work environment and cognitive skills required between esports and pilots, air traffic controllers, and military drone operators for example^33^. This framework could be extended to study how ability level on a task moderates the effect of a given intervention on task performance.

1v1 Rocket League in particular is an ideal esport to use as a performance task in an experimental setting as it has a short & predictable match length (5–10 min), allowing for many trials within an experimental setting, a simpler experimental design than other esports due to the ability for one to play individually, and experimenters can easily access player rank and in-match metrics. The results of our analysis specifically inform as to the important PIs of interest when evaluating the efficacy of an experimental intervention on Rocket League performance. A reduction of the outcome variable, GD, alongside key PIs such as *difference in shots taken, difference in saves made, and difference in time spent goalside of the ball*, would represent a negative effect on the intervention on task performance. Interestingly, a reduction in *time spent at supersonic speed* or *instances of powerslides* following an intervention, but a maintenance of performance, could suggest an adaptation by players to simplify their play style to maintain performance following an intervention.

This is the first study to use Random Forest models to identify PIs within an esport. Random Forests are robust to data of any distribution from a large number of features (regardless of if many are actually predictive of the outcome or not) and can ascertain non-linear effects and complex interactions without prior specification. Thus, Random Forests present as a valuable tool for notational analysis within esports, which is in its infancy and has limited prior information available on potential PIs for various games and genres. Random Forests for notational analysis in esports could be used to explore what predictor metrics are most important for match outcome in other genres, such as FPS’s and MOBA’s.

### Limitations and future research

When considering the power of Random Forests as a notational analysis, one limitation is that feature importance measures from Random Forest models can show bias when features are correlated^34,35^. To mitigate this, where the variance of one predictor metric could be entirely explained by one or more other metrics, these additional metrics were removed, and multicollinearity was assessed for each model with multicollinear metrics being removed. Additionally, features shown to be important for game outcome or skill within each model showed no greater correlation with other features compared to those not found to be important (correlation matrices for all models can be found in Supplementary Figs. S1–S10). Future research should consider feature importance measures such as permutation conditional on remaining features^34^, “leave-one-covariate-out”^36^, and “permute and relearn”^35^ to address correlated features, however given the large amount of data and extra computational resources required for these methods, they were not feasible here.

In this study, we chose to exclusively explore 1v1 Rocket League. While identical in game mechanics, positioning and decision making vary between 1v1, 2v2, and 3v3 formats of Rocket League. Hence, PIs and RIs for team-based Rocket League may be different to 1v1. However, this analysis would have been greatly complicated if we additionally included team-based Rocket League, as interactions between teammates would have to be considered, further complicating analysis^29^. Interestingly, 1v1 is considered by many professional Rocket League players (i.e., “Flakes”) to be the best way to improve in Rocket League overall due to affording players more time to interact with the ball compared to other formats. Hence, the PIs and RIs here can provide great benefit for all Rocket League players and coaches, even if improvement specifically in 1v1 Rocket League is not the primary goal. However, future research should attempt to use similar analysis methods to those described here to identify the PIs and RIs for 2v2 or 3v3 Rocket League.

## Conclusions

In summary, this study is the first to use Random Forest models to identify PIs and RIs that could predict match outcome and rank respectively across over 20,000 matches in the rapidly emerging esport of Rocket League. Overall, spending more time goalside of the ball, *taking more shots*, *conceding less shots*, and *making more saves*, were all identified as beneficial for in-match performance across all ranked matches. All metrics were found to be significantly important (and thus, RIs) for a Random Forest model’s ability to predict player rank, and we have classified the order of importance of these metrics using our model. Interestingly, we found that *time spent at supersonic speed*, *time spent on the ground*, *shots conceded*, and *time spent goalside of the ball* were the most important RIs. This type of analysis can provide useful insight to Rocket League players and coaches regarding the structuring of VPT programs to improve match success of in-game rank. The findings from our analysis also provides researchers with key metrics to consider if using Rocket League as a performance task in experimental research.

## Methods

Data from 33,854 total matches were downloaded from “www.ballchasing.com” (http://www.ballchasing.com), a repository of Rocket League match replays and statistics, on 16/12/2020. In addition to downloading all the data for all Bronze (4111 matches) and GC matches (9743 matches), we downloaded all the data for the most recent 10,000 Gold and Diamond rank matches respectively. Data were gathered from matches prior to September 29, 2020, and this was done for two reasons. Firstly, an update to the game with an accompanied rank redistribution saw additional ranks added after this date. Secondly, this update hindered the ability for “www.ballchasing.com” to recognise the ranking of players within a match. These issues have since been resolved, however were such during our data collection and analysis that we did not include match data from after September 29, 2020. These data were downloaded directly from the public domain, are freely available to all individuals, and results are completely de-identified. Further, all General Data Protection Regulations (GDPR) have been fulfilled.

### Data processing

Using the website’s inbuilt filters and replay group function, match statistics were downloaded as a .csv file. Each match file contained general descriptions of the match (i.e., map, player names, cars used) as well as 65 columns corresponding to data describing the performance for 65 in-match metrics (potential performance (PIs) and rank (RIs) indicators) (Supplementary Data S1 contains an anonymised sample file directly from “www.ballchasing.com”).

From here, many processing steps were undertaken to result in the final 28 “raw-score” metrics and 26 “difference-score” metrics included in the Random Forests analyses (see Table 1). We have provided a brief description of these steps below, however the reader is directed to the Supplementary Methods where we provide a detailed description of these steps, allowing for reproduction.

First, we calculated match length using metrics provided, and used this to normalise all metrics that were not already presented as a percentage of match length to the average length of a rocket league match (360s). Second, we removed all ‘draws’ in the data, as well as matches that did not exceed 150 s duration to avoid overestimation of time normalised data. Next, we recalculated average speed using these time measures, and used metrics provided to calculate the metric ‘True boost wastage’. True Boost Wastage represents the proportion of “boost” used when a player is already travelling at max or near max speed. It is generally considered a measure of poor “boost” use, or wasted “boost”^37,38^. The Supplementary Methods contain descriptions for “boost”, true boost wastage and all other metrics are described in greater detail.

From here, we calculated “difference-scores” for each metric (the difference between a given player and their opponent’s metric values). This was done in light of evidence that difference-scores can provide superior predictive ability compared to “raw-score” metrics in a Random Forest analysis of PIs in Rugby Union^9^. We then maximised independence of data by removing all games besides the most recent ten from a given player, and de-identified the data. Penultimately, we ensured that no metrics could be combined to entirely explain the variance of another included metric. Lastly, shots conceded difference and demos taken difference were removed, as these metrics mirrored shots taken difference and demos inflicted difference metrics respectively (see Supplementary Methods).

Following the above processing steps, 28 raw-score predictor metrics and 26 difference-score predictor metrics were retained per match. “Raw-score metrics” and “difference-score” metrics were split into two in separate dataset files and metrics in each file were divided into four categories, offense/defence metrics, boost metrics, player movement metrics and player positioning metrics (see Table 2).

**Table 2:** Predictor metrics obtained through “www.ballchasing.com” and subsequent processing.

Offense/defense	Boost	Movement	Positioning
Shots taken	Boost used	Average speed	Time spent on the ground
Shots conceded^†^	Average boost reserve	Time spent at "slow speed"	Time spent high in the air
Demos inflicted	Total boost collected	Time spent at "supersonic speed"	Time spent goalside of the ball
Demos taken^†^	Count boost collected from big pads	Average duration for a powerslide	Time spent in the defensive third
	Count boost collected from small pads	Instances of powerslides	Time spent in the offensive third
	Total boost stolen		
	Count boost stolen from big pads		
	Count boost stolen from small pads		
	True boost wastage (%)		
	Total boost overfill collected		
	Total boost overfill stolen		
	Time spent at 100 boost		
	Time spent at 0 boost		

Metrics are time normalised to an average match length (360 s) unless provided as a percentage of total time in the original dataset, Metrics are expressed both as “raw-score” and “difference-score” except those denoted by a ^†^, which are “raw-score” only.

### Analysis 1: identifying a continuous outcome measure

Upon identifying the relevant matches and metrics to carry forward for analyses, and in line with the first aim of this study, we determined a continuous *match outcome* metric that that could be reasonably substituted for the binary win vs. loss outcome measure while providing additional information regarding the severity of a win or loss. To do this, the *in-game score difference* (IGSD) and *goal difference* (GD) metrics were considered as candidates. Point-beserial correlations were conducted between the candidate measures and the dichotomous “win vs. Loss” (WL) metric across all rank groups and with matches from all ranks combined. Additionally, we explored the accuracy of the two candidate metrics in separating WL, using zero as the cut-off.

### Analysis 2: obtaining performance indicators (PIs)

Our second objective was to identify the metrics that best predicted our match outcome measure (GD) within matches across individual rank groupings, and within matches across all included ranks combined (PIs). To address this objective, individual Random Forest regression models were created each for matches within given ranks (i.e., Bronze matches only) and for all matches, regardless of rank. Two models were created per rank (and with all matches combined); one using raw-score metrics and one using difference-score metrics. Random Forest regression models were created using the statistical software, R: A Language and Environment for Statistical Computing (Vienna, Austria).

In addition to the steps taken in data processing to remove metrics that, when combined, could entirely account for the variance of another metric, multicollinearity was assessed for each dataset using qr-matrix decomposition (*p* < 0.05) in the rfUtilities package in R^39^. Average speed within the model with GC matches only was identified as multicollinear and was subsequently removed from further analyses.

Random Forest models were then created using the randomForest package in R^40^. The sole purpose of these models was to determine the optimal value of ntree for each model (amount of CARTs within the Random Forest model). The optimal ntree was the number under 1000 that gave the lowest mean square error of GD, provided the mean square error in the number of trees surrounding this number was also stable. Mean square error was measured using out-of-bag (OOB) data; that is, using only matches that were not involved in the creation of a given tree within the forest. A maximum of 1000 trees was chosen as it was likely that this would be sufficient to produce highly predictive models if this was possible given the data (default is 500) while simultaneously balancing computational speed. The default mtry value was used, due to evidence that the default values provided within the RandomForest package perform well^40^, and that this number does not tend to affect the performance of the model greatly^25,27^.

Using the optimal ntree, new Random Forest models were then created using the rfPermute package in R^41^. As well as making a Random Forest model, the rfPermute package provides significance values for metric importance. The percentage increase in mean square error (%incMSE) observed when a metric is permuted compared to when no metrics are permuted was used as the measure of metric importance score for each metric. %incMSE was chosen over Mean Decrease in Impurity (Gini), as Gini has shown to be biased when the scale that features are measured on varies^42^. To obtain a significance value, rfPermute additionally permutes the outcome metric (GD) a specified number of times, so that there is to be no relationship between any predictor metric and GD. Significance values are obtained per predictor metric each time GD is permuted, forming a “null distribution” of importance scores per predictor metric. P-values are then calculated from the fraction of metric importance scores within this “null distribution” that are greater than the metric importance score obtained when GD was not permuted, with *p* < 0.05 being considered a significant metric.

### Analysis 3: obtaining indicators of in-game rank (RIs)

The third objective of this research was to identify the metrics that were able to predict the rank of players within a match regardless of match outcome (i.e., win vs. loss, IGSD & GD). To do so, a Random Forest *classification* model was created in R using data from all included ranks. Unlike a regression model, which provides a numerical outcome prediction, a Random Forest classification model provides a categorical prediction. Feature dependence was explored in the same manner as in Analysis 2. For metric importance, GD was permuted 50 times.

Raw-score mean decrease in accuracy (MDA) was chosen as the measure of metric importance over Mean Decrease in Impurity (Gini) and normalised MDA, for the same reasons as mentioned for the regression models and %incMSE. A Random Forest classification model was only created using raw-score metrics because difference-score metrics should always tend to approach 0 when not considering match result.

**Figure 6:**
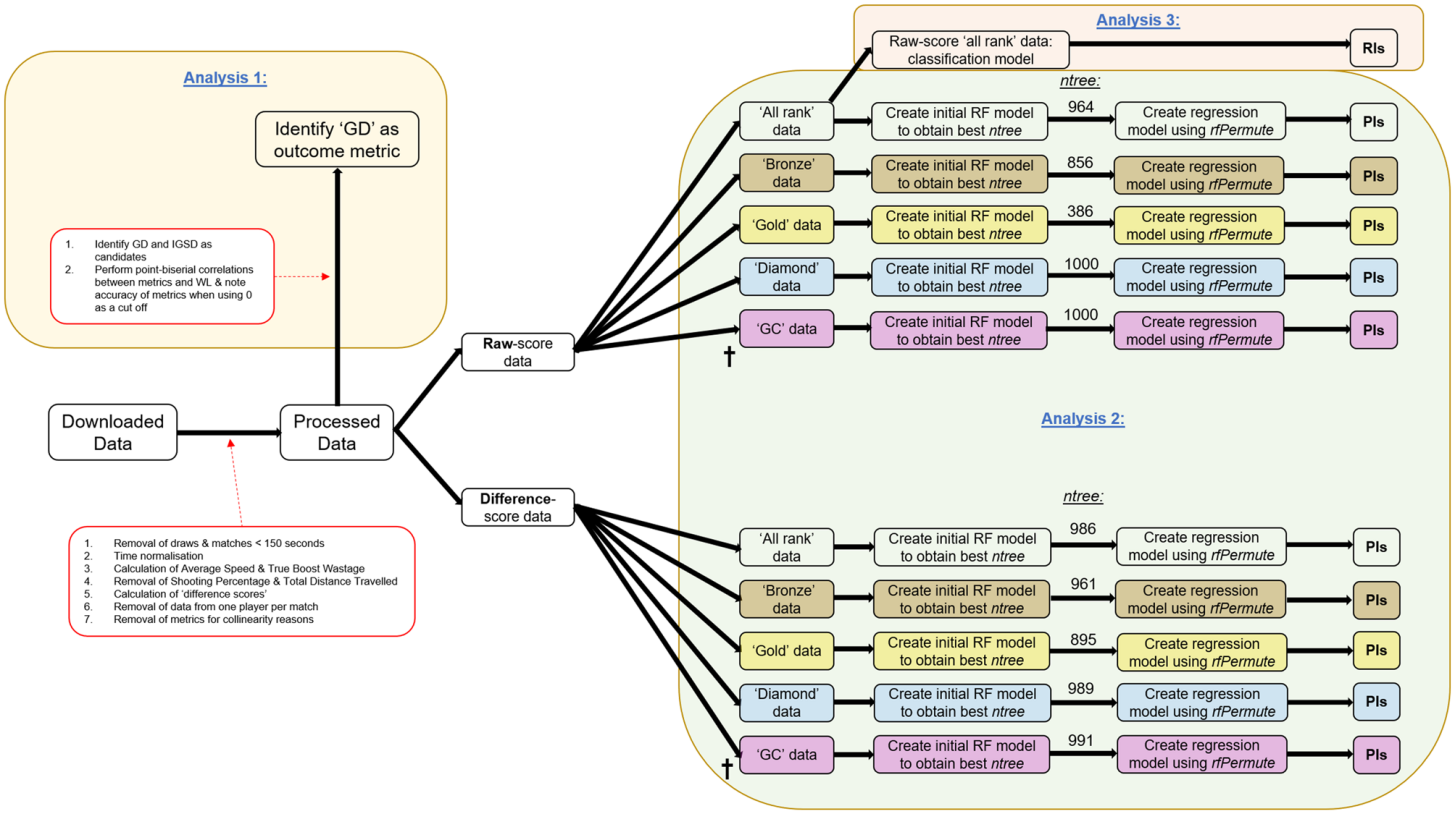
Flowchart depicting the methods of the current study. The three outlined analyses are labelled in blue. The ^†^highlights where average speed was removed due to multicollinearity.

A flowchart outlining the methods for this study can be found in Fig. 6.

## Supplementary Information

Supplementary Information 1.

Supplementary Figures.

Dataset S1.

## Data Availability

The datasets generated using the methods described and which undertook the described analysis in the current study are openly available in osf.io and can be found at 10.17605/OSF.IO/J5NM2.
