# Supplementary material for: A Random Forest approach to identify metrics that best predict match outcome and player ranking in the esport Rocket League
Source: Sci Rep. 2021 Sep 29;11:19285. doi: 10.1038/s41598-021-98879-9 (PMC8481284; doi:10.1038/s41598-021-98879-9)
Supplement: Supplementary file 1 — Supplementary Information 1. [file 41598_2021_98879_MOESM1_ESM.docx]

**A Random Forest approach to identify metrics that best predict match outcome and player ranking in the esport Rocket League**

### Supplementary Methods - Detailed Description of Processing Steps

The following describes the processing steps applied to these metrics, resulting in the 28 “raw-score” metrics and 26 “difference-score” metrics included in the Random Forests analyses (see Table 1).

1. Matches that resulted in a “Draw” (most likely resulting from automatically saved and uploaded replay files from matches that did not take place due to one player not connecting to the server, as draws cannot otherwise occur in Rocket League) were removed
2. Matches with a duration < 150 seconds to avoid overestimation of time normalised data
3. For the remaining matches (30,639 total), match length was calculated five different ways per match (ML1-ML5), using the following five different combinations of metrics that, when combined, covered the entirety of the match:

$ML1= time spent at slow speed + time spent at boost speed + time spent at supersonic speed$

$ML2 = time spent on the ground + time spent low in the air + time spent high in the air$

$ML3 = time spent in front of the ball + time spent goalside of the ball$

$ML4 = time spent in the defensive half + time spent in the offensive half$

$ML5 = time spent in the defensive third + time spent in the neutral third + time spent in the offensive third$

The average (MLi) of ML1-ML5 was used as rounding discrepancies in the downloaded data could result in variance of up to 0.05sec depending on the combination of metrics used. Further, this average value varied by 3.45±6.96 seconds for different players within the same match, hypothesised to resemble the discrepancy in initial match loading time (commonly seen as players can play one another on different platforms), however this is uncertain. Given this small discrepancy, we calculated the mean match length for a given match from the match lengths calculated for each player, and then assigned that value to both players within the match (ML; see equations below). $MLi = \frac{ML1+ML2+ML3+ML4+ML5}{5}$ $Match Length \left( ML \right)= \frac{MLi\left( player \right)+MLi(opponent)}{2}$

The calculation of ML described above was performed for all matches and resulted in an overall average all match duration of 360.45 seconds. We thus normalised all metrics not already expressed as a percentage to an average match length of 360 seconds, using the formula below:

$Normalised Metric= \frac{Metric value}{ML} \times360$

1. After these calculations and time normalisation, we excluded the shooting percentage metric as its calculation (percentage of goals scored from all the shots taken) directly related to our outcome variable (GD).
2. *Average speed* was recalculated using the *total distance travelled* (TDT) metric and ML using the following equation.

$Average speed= \frac{TDT}{ML}$ (9)

1. Following the calculation of *average speed*, we excluded the *total distance travelled* metric from further analyses.
2. We calculated an additional metric called *true boost wastage*, using *total boost used while supersonic* and *total boost used* metrics. *True boost wastage* represents the proportion of “boost” used when a player is already travelling at max or near max speed. It is generally considered a measure of poor “boost” use, or wasted “boost” ^37,38^. Supplementary file 2 contains descriptions for “boost”, true boost wastage and all other metrics are described in greater detail. True boost wastage was calculated using the following equation

$$True boost wastage= \frac{total boost used while supersonic}{total boost used} \times100$$

3. Data from five players (two Bronze, two Gold, one GC) were removed due to using no “boost” throughout a match, resulting in no *true boost wastage* metric value.
4. Upon identifying and calculating all the metrics to be included for subsequent analysis, we also calculated “difference-scores” for each metric (the difference between a given player and their opponent’s metric values). Difference scores were as per the following:

$Difference score for metric{{}^{'}x}^{'}= players 'x'- opponents 'x'$

5. To eliminate the representation of matches twice in the same dataset, the data from one player in each match were removed (50%).
6. In many cases, certain players contributed many more matches than others (i.e., in the GC rank, one player contributed 812 matches). In order to maximise the independence of all the matches and data within a rank, only the ten most recent matches from the same in-game account name were retained (though the same account name could appear more than ten times as an opponent), with a maximum of one match retained for the same player-opponent combination. This strategy was chosen to both maximise the independence of the data while also maintaining a substantial amount (>45%) of matches for each rank (2527 Bronze, 7226 Gold, 7193 Diamond & 4643 GC*****).
7. Following this, in-game account name was removed to de-identify the data. Thus, from this point onwards, it was impossible to identify individuals from the data.
8. When one or more metrics were able to entirely explain the variance of another included metric, this was considered problematic from a collinearity standpoint, and so certain metrics were removed to mitigate this. Where this occurred, the “middle” metric was excluded (i.e., time spent in the neutral third was removed where time spent in the attacking and defensive thirds were retained; or time at boost speed was excluded where time spent at slow and supersonic speeds were retained). Furthermore, where positioning metrics were provided as “percentage spent in thirds” and “percentage spent in halves”, those expressed in “halves” were removed. Also, total boost collected/stolen from big and small pads were removed in favour of retaining ‘total boost collected/stolen’, as values for boost collected/stolen for big pads and small pads was also provided as a ‘count’ (which does not necessarily correspond to ‘total’ within Rocket League) as well. Overall, 24 metrics were removed in this way. These metrics are listed below.
   1. Raw and difference score metrics for: Total boost collected from big pads, total boost collected from small pads, total boost stolen from big pads, total boost stolen from small pads, total boost used while supersonic, time at boost speed, total time of powerslides, time low in the air, time in front of the ball, time in defensive half, time in offensive half, time in neutral third.
9. Finally, *shots conceded difference* and *demos taken difference* were removed, as these metrics mirrored *shots taken difference* and d*emos inflicted difference* metrics respectively (see supplementary file 2 for elaboration).

### Supplementary Methods - Description of Metrics

### Offense/Defence:

Shots taken/conceded: A ‘shot’ is when a player hits the ball and the ball gets within close proximity to the opponents net.

Demos inflicted/taken: A ‘demo’ is inflicted when a player drives into an opponent at max or near max speed and at the correct angle, removing the opponent for three seconds before they respawn at one of two locations within the defensive third. A ‘demo’ is taken when the opponent player demo’s the player of interest.

### Boost:

Boost is a finite resource present in Rocket League which, when used, allows for rapid car acceleration and greatly assists in the ability to get high in the air. Boost can be collected from big pads (6 locations, provide 100 boost and respawn every 10 seconds) or small pads (28 locations, provide 12 boost and respawn every 3 seconds), which are in a standardised location on each map. A player can only have a maximum of 100 boost at any given time.

Boost used: The total amount of boost used over the course of a match.

Average boost reserve: The average amount of boost a player has spare throughout the match.

Total boost collected: The total amount of boost collected over the course of a match.

Count boost collected from big/small pads: The total amount of times a player gained any amount of boost from a big/small pad.

Total boost stolen: The total amount of boost collected specifically from the opponents half of the field.

Count boost stolen from big/small pads: The total amount of times a player gained any amount of boost from a big/small pad in the opponents half of the field (excluding boost on the halfway line).

True boost wastage (%): The proportion of total boost used while at max or near max speed (i.e. supersonic speed)

Total boost overfill collected/stolen: If a player collects 100 boost from a big pad while they currently have boost spare, the amount of boost they had spare would be considered their ‘overfill’.

Time spent at 0/100 boost: The total amount of time a player spends with either full boost or no boost over the course of a match.

### Movement:

Average Speed: The average speed (in ‘unreal units per second’) that a player travels at over the course of a match.

Time spent at ‘slow’ speed: The total time a player spends travelling at a speed less than the max speed obtainable using only the accelerator (~60% of max attainable speed).

Time spent at ‘supersonic’ speed: The total time a player spends travelling at 95-100% of max attainable speed.

Average duration for a powerslide: ‘Powersliding’ is a mechanic in Rocket League that allows a player to (a) turn sharper and (b) conserve momentum when landing, particularly when landing in an orientation that is different to the momentum of the players’ car. ‘Average duration for a powerslide’ refers to the average time in which a powerslide is held when a player chooses to use this mechanic.

Instances of powerslides: Number of times a player uses the powerslide mechanic within a match.

### Positioning:

Time spent high in the air: Total amount of time in which all four wheels of a players car are grounded throughout a match.

Time spent high in the air: Total amount of time in which a players car is above the height of the players goal.

Time spent goalside of the ball: Total amount of time in which a players car is on the defensive side of the ball (i.e. between the ball and the goal they are protecting).

Time spent in the offensive/defensive third: Total amount of time in which a players car is within the third of the playing area that includes the opponents goal (offensive third) or own goal (defensive third).
