# Supplementary material for: A Random Forest approach to identify metrics that best predict match outcome and player ranking in the esport Rocket League
Source: Sci Rep. 2021 Sep 29;11:19285. doi: 10.1038/s41598-021-98879-9 (PMC8481284; doi:10.1038/s41598-021-98879-9)
Supplement: Supplementary file 2 — Supplementary Figures. [file 41598_2021_98879_MOESM2_ESM.docx]

**A Random Forest approach to identify metrics that best predict match outcome and player ranking in the esport Rocket League**

### Supplementary Figures S1-10

Each of the following are correlation matrices created between the predictor metrics in each regression model created. Numbers are indicative of strength of correlation (*r* value) and are only presented if *p* < .001.

**Raw Score Metrics:**


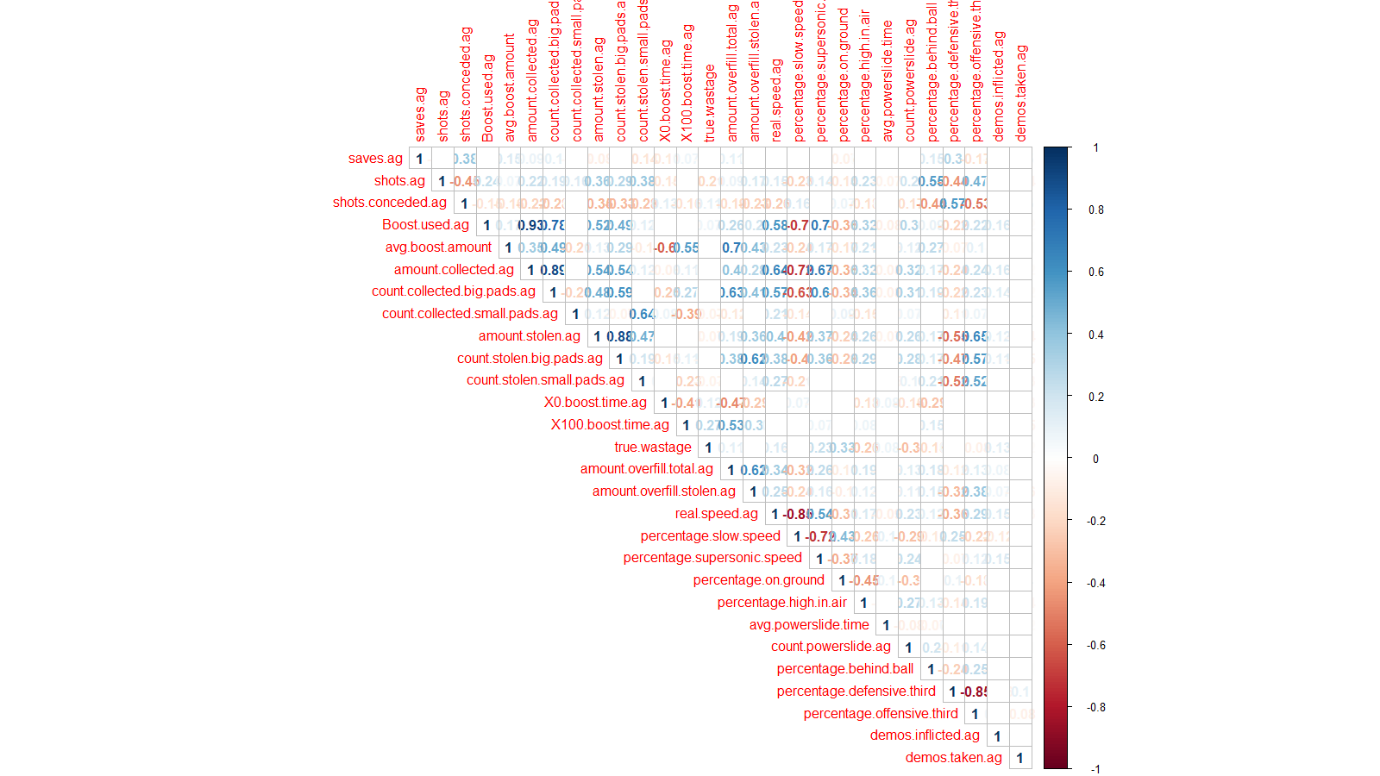


Figure S1: Correlation matrices of all predictor metrics within the model created using raw-score metrics and data from bronze rank matches.


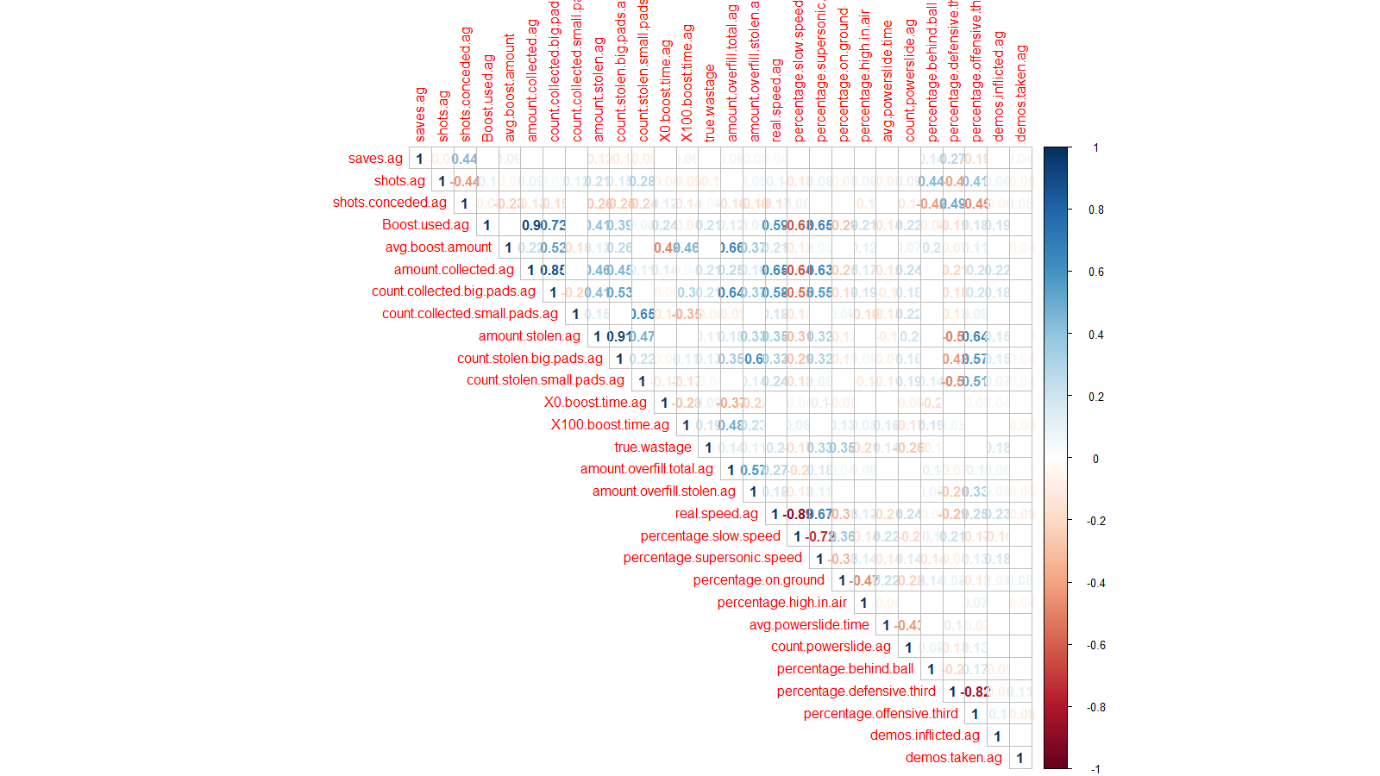


Figure S2: Correlation matrices of all predictor metrics within the model created using raw-score metrics and data from gold rank matches.


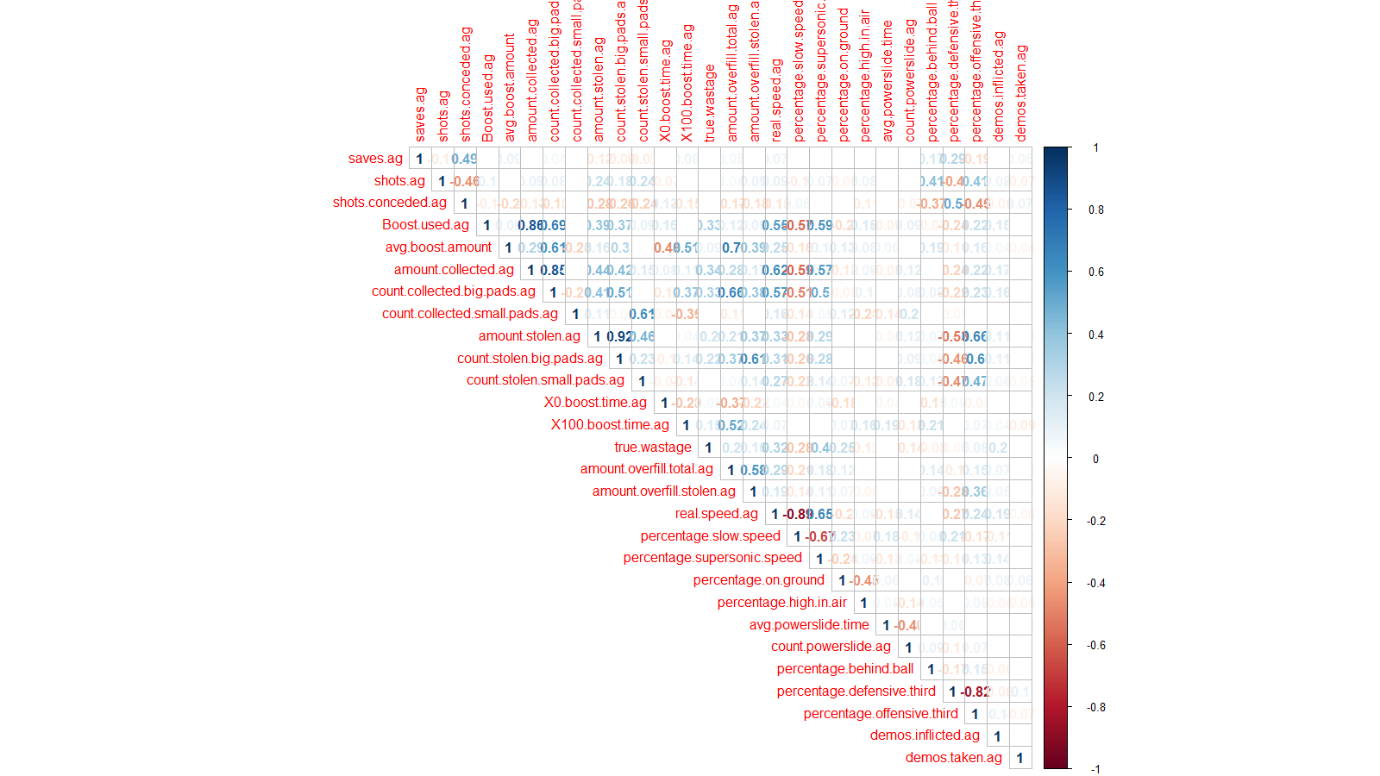


Figure S3: Correlation matrices of all predictor metrics within the model created using raw-score metrics and data from diamond rank matches.


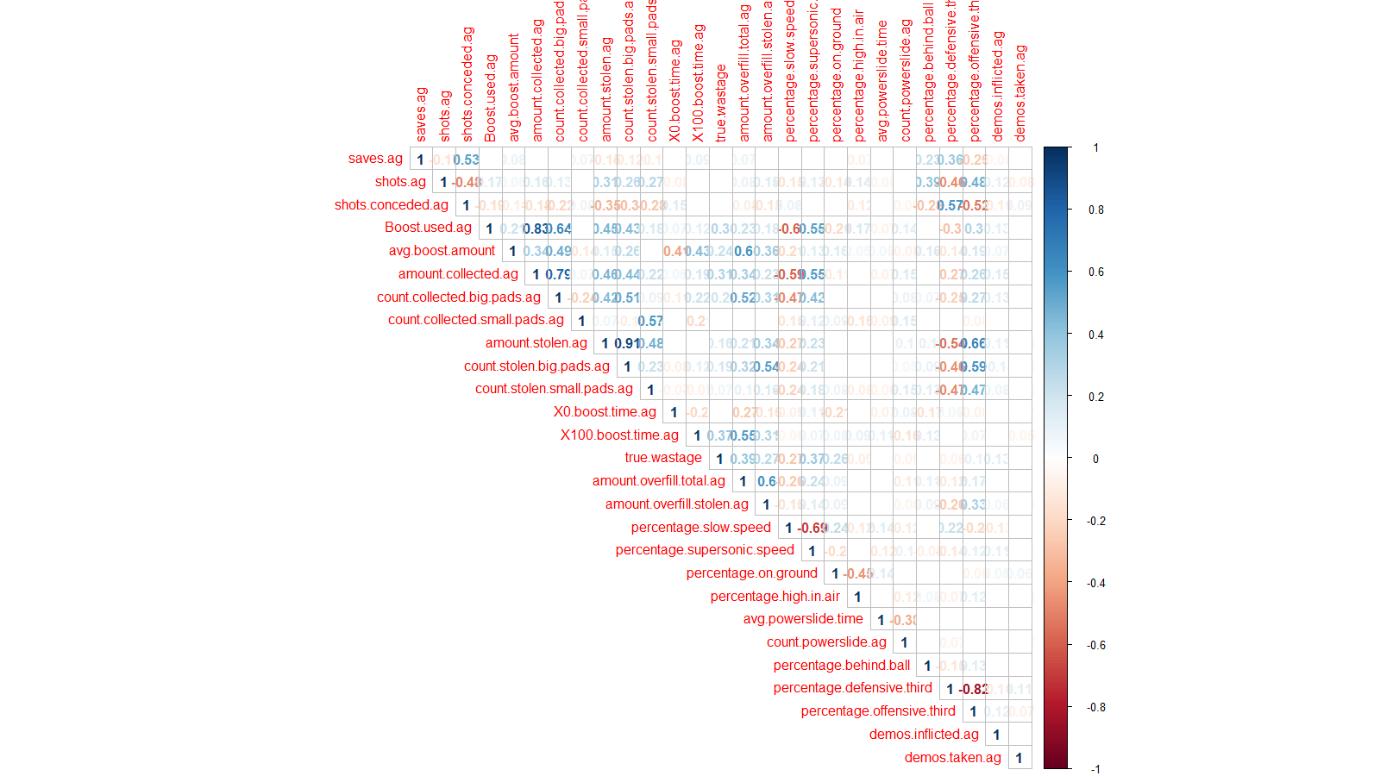


Figure S4: Correlation matrices of all predictor metrics within the model created using raw-score metrics and data from GC rank matches.


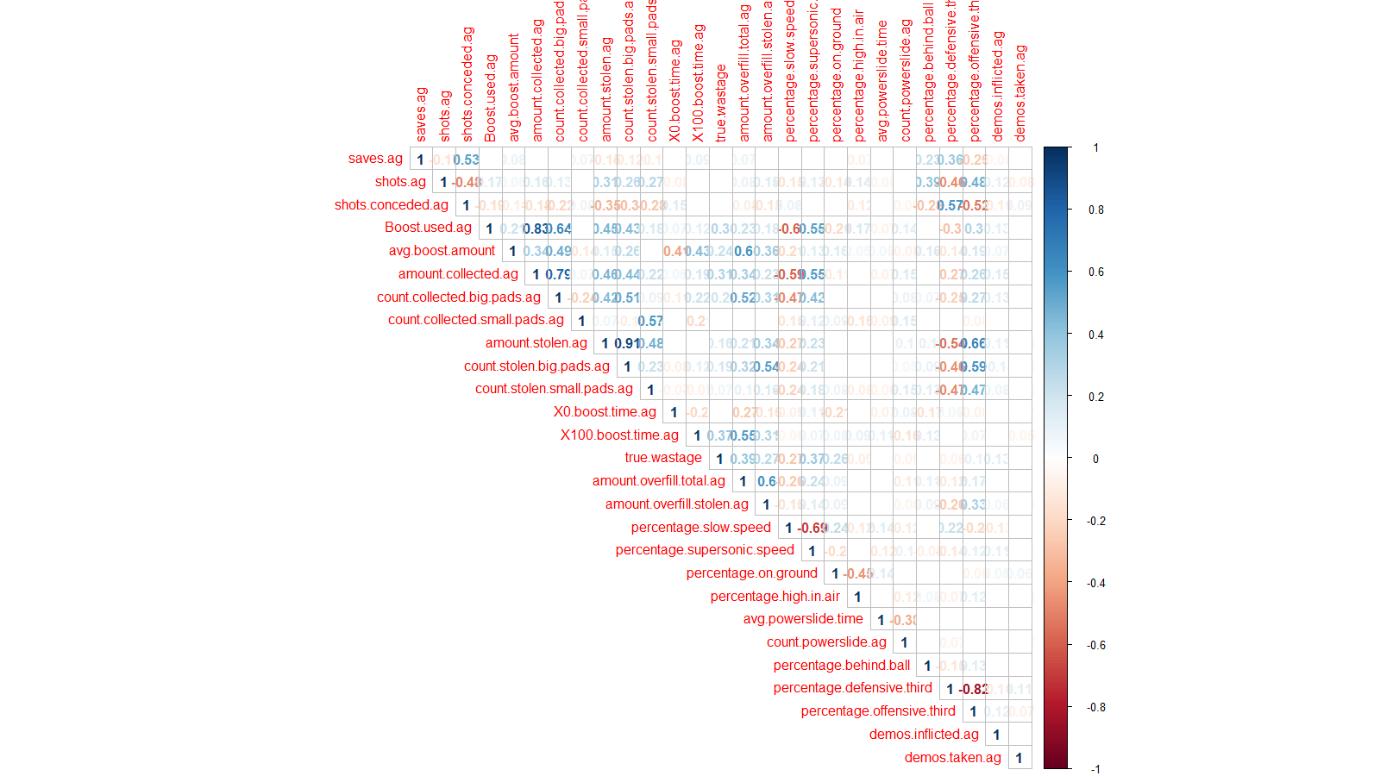


Figure S5: Correlation matrices of all predictor metrics within the model created using raw-score metrics and data from matches of all ranks.

**Difference Score Metrics:**


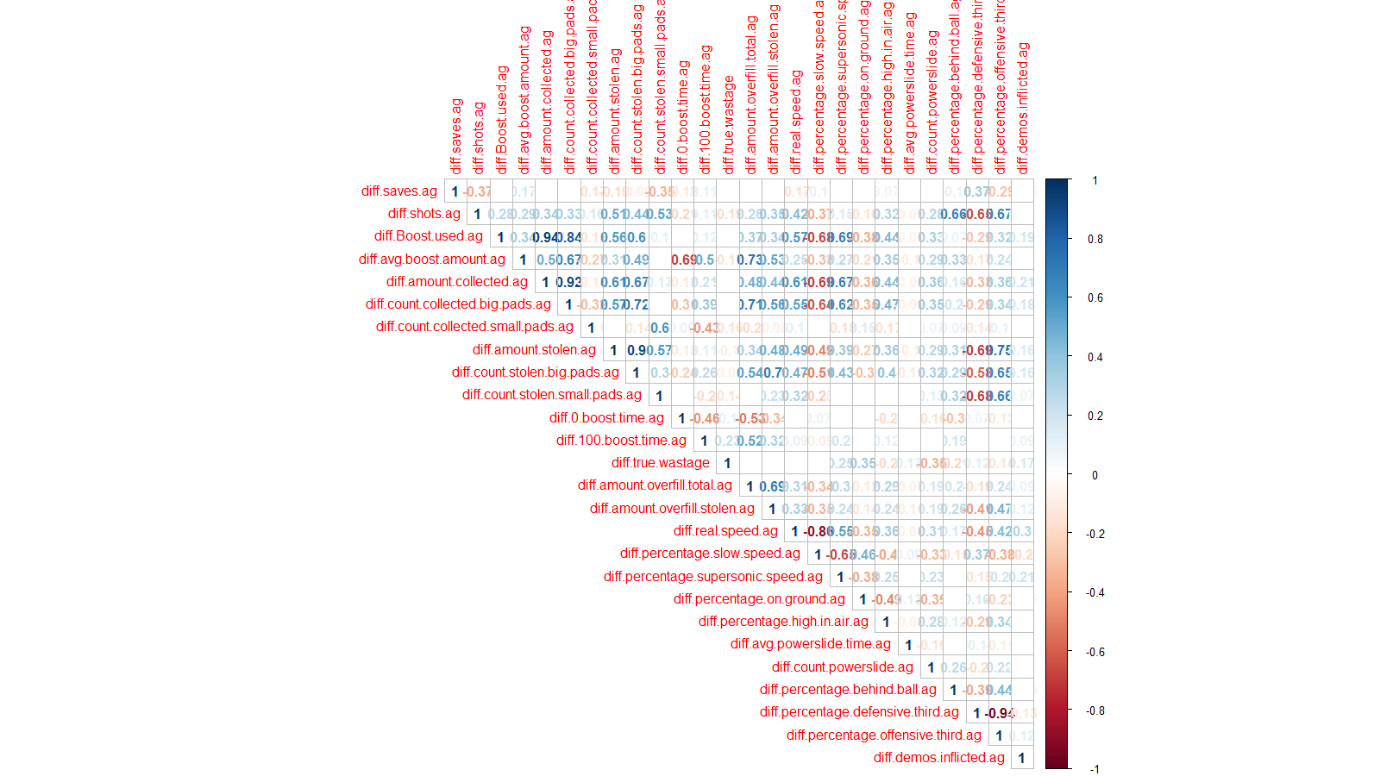


Figure S6: Correlation matrices of all predictor metrics within the model created using difference-score metrics and data from bronze rank matches.


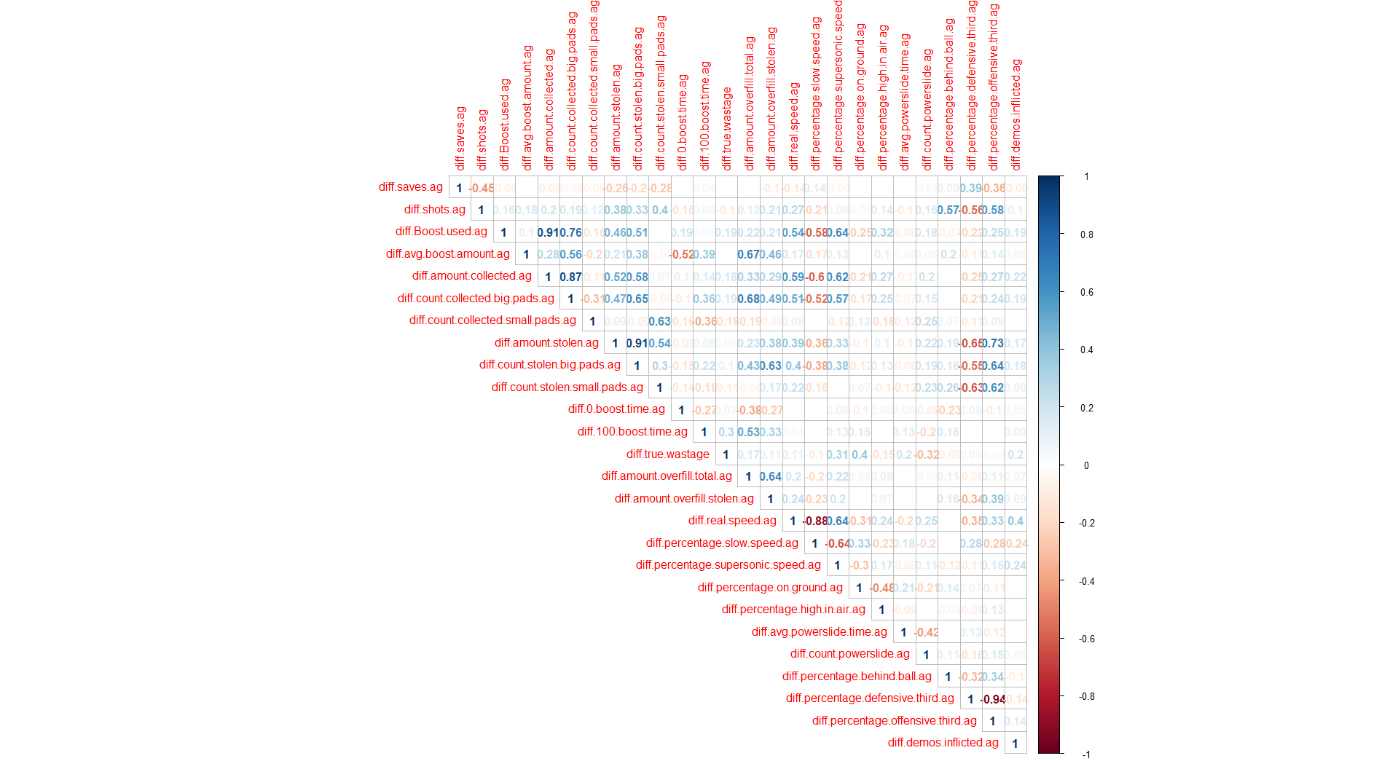


Figure S7: Correlation matrices of all predictor metrics within the model created using difference-score metrics and data from gold rank matches.


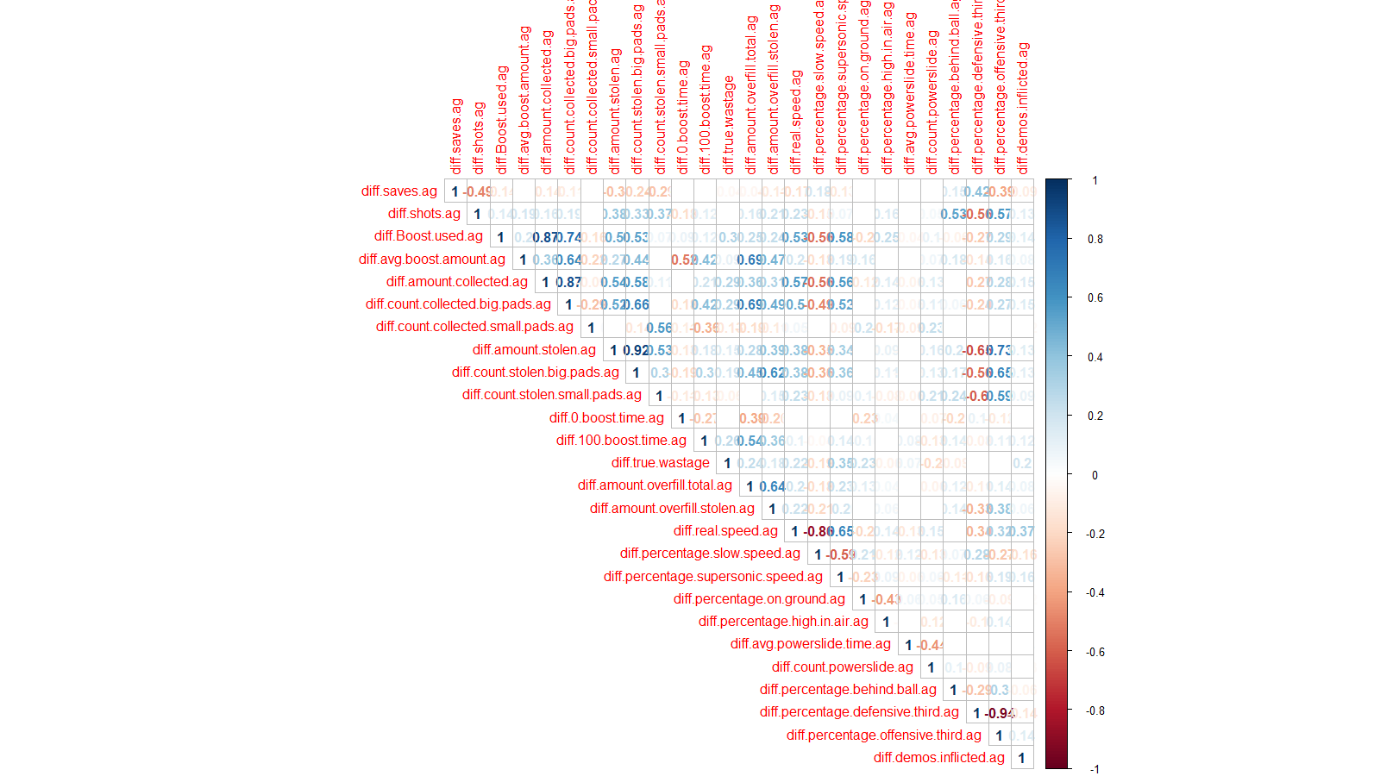


Figure S8: Correlation matrices of all predictor metrics within the model created using difference-score metrics and data from diamond rank matches.


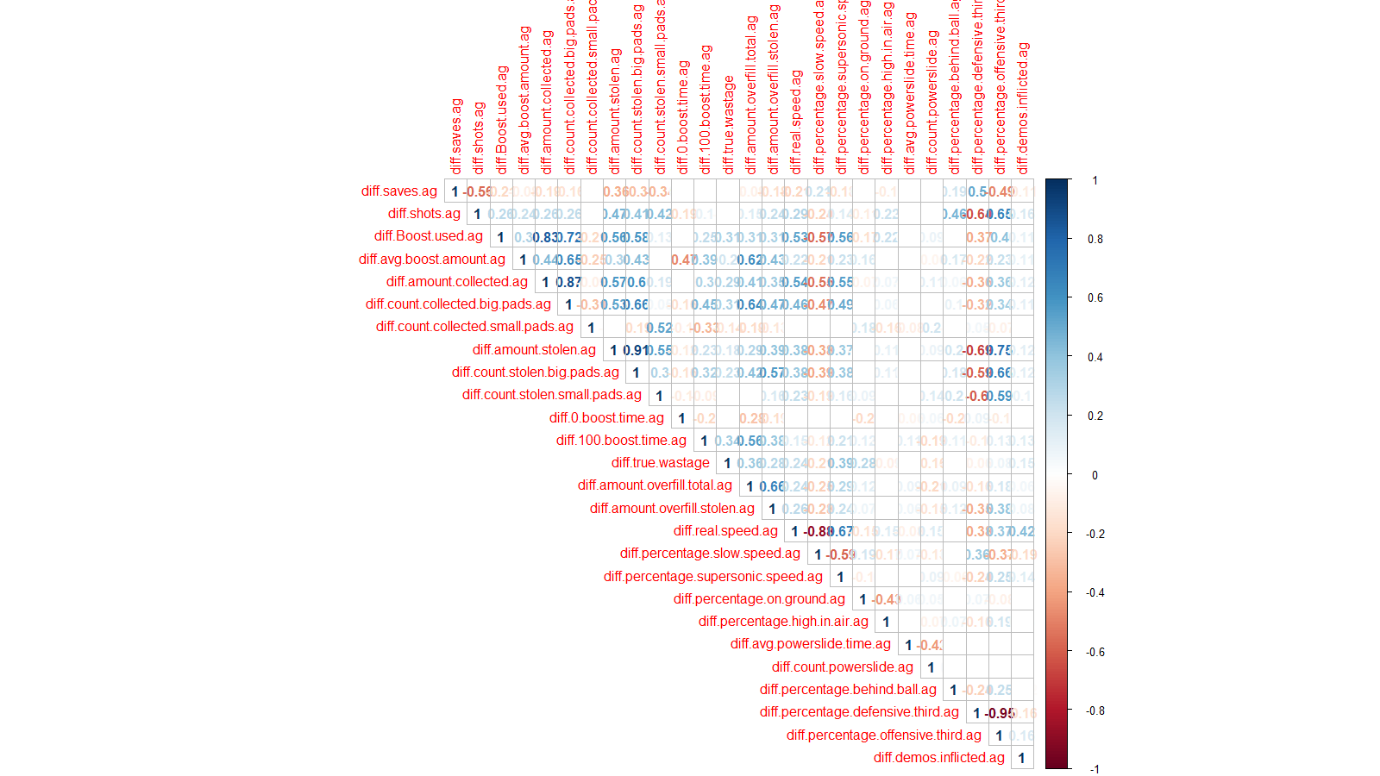


Figure S9: Correlation matrices of all predictor metrics within the model created using difference-score metrics and data from GC rank matches.


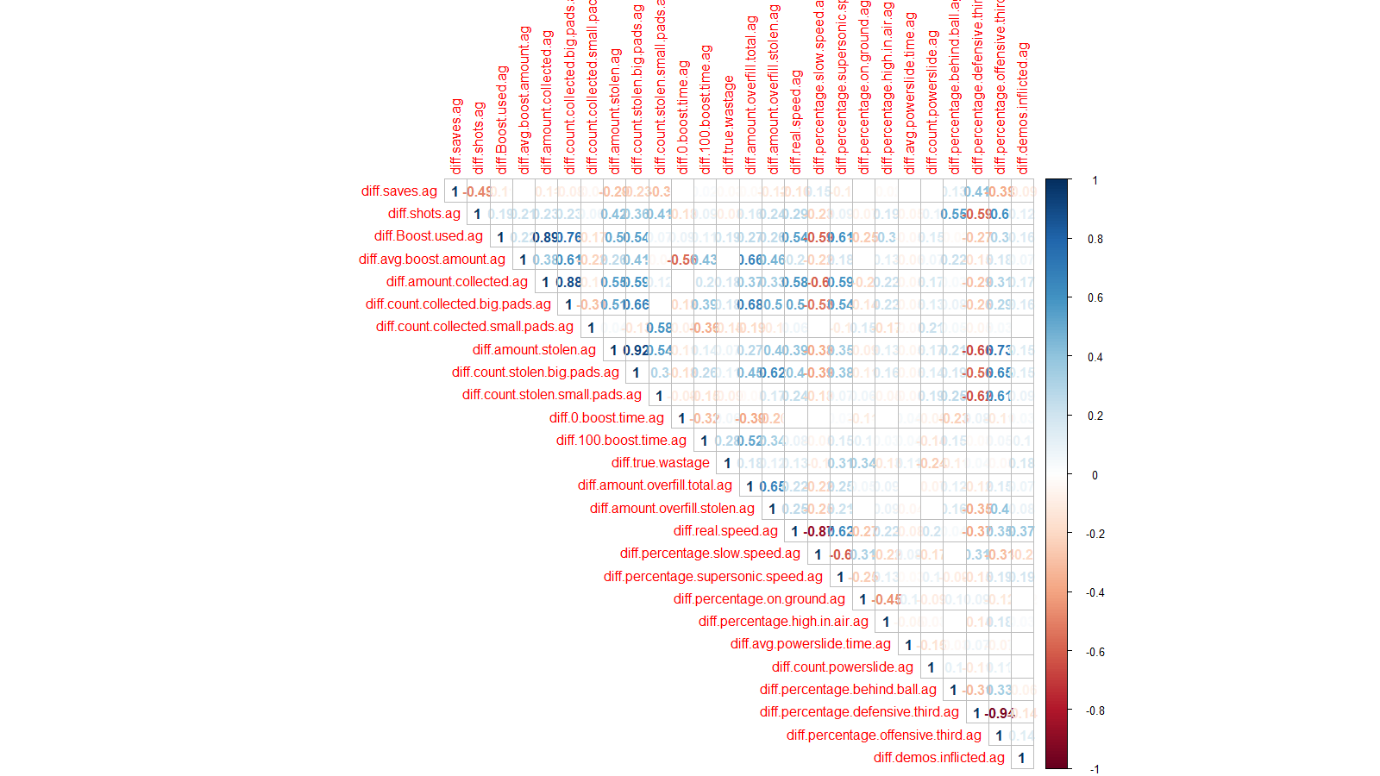


Figure S10: Correlation matrices of all predictor metrics within the model created using difference-score metrics and data from matches of all ranks.
